# High-Frequency Head Impact Disrupts Hippocampal Neural Ensemble Dynamics

**DOI:** 10.3389/fncel.2021.763423

**Published:** 2022-01-18

**Authors:** Daniel P. Chapman, Stephanie S. Sloley, Adam P. Caccavano, Stefano Vicini, Mark P. Burns

**Affiliations:** ^1^Georgetown Interdisciplinary Program in Neuroscience, Georgetown University Medical Center, Washington, DC, United States; ^2^Department of Pharmacology and Physiology, Georgetown University Medical Center, Washington, DC, United States; ^3^Department of Neuroscience, Georgetown University Medical Center, Washington, DC, United States

**Keywords:** calcium imaging, subconcussive head impact, mouse model, brain injury–traumatic, plasticity

## Abstract

We have recently shown that the cognitive impairments in a mouse model of high-frequency head impact (HFHI) are caused by chronic changes to synaptic physiology. To better understand these synaptic changes occurring after repeat head impact, we used Thy1-GcCAMP6f mice to study intracellular and intercellular calcium dynamics and neuronal ensembles in HFHI mice. We performed simultaneous calcium imaging and local field potential (LFP) recordings of the CA1 field during an early-LTP paradigm in acute hippocampal slice preparations 24 h post-impact. As previously reported, HFHI causes a decrease in early-LTP in the absence of any shift in the input-output curve. Calcium analytics revealed that HFHI hippocampal slices have similar numbers of active ROIs, however, the number of calcium transients per ROI was significantly increased in HFHI slices. Ensembles consist of coordinated activity between groups of active ROIs. We exposed the CA1 ensemble to Schaffer-collateral stimulation in an abbreviated LTP paradigm and observed novel coordinated patterns of post stimulus calcium ensemble activity. HFHI ensembles displayed qualitatively similar patterns of post-stimulus ensemble activity to shams but showed significant changes in quantitative ensemble inactivation and reactivation. Previous *in vivo* and *in vitro* reports have shown that ensemble activity frequently occurs through a similar set of ROIs firing in a repeating fashion. HFHI slices showed a decrease in such coordinated firing patterns during post stimulus ensemble activity. The present study shows that HFHI alters synaptic activity and disrupts neuronal organization of the ensemble, providing further evidence of physiological synaptic adaptation occurring in the brain after a high frequency of non-pathological head impacts.

## Introduction

Traumatic brain injury (TBI) is one of the most common neurological disorders worldwide. The majority of TBIs (∼80%) are mild TBI with symptom resolution occurring in a matter of days to weeks (Cassidy et al., 2004; Laker, 2011; Frost et al., 2013; Voss et al., 2015; Blennow et al., 2016; Lefevre-Dognin et al., 2021). Repetitive mild TBI (rmTBI), such as those injuries seen in contact sports athletes and members of the armed forces, increases the severity and duration of symptoms (Guskiewicz et al., 2007; Greco et al., 2019). Evidence is also emerging to suggest that sub-concussive impacts such as heading a soccer ball or sustained high frequency low amplitude cranial movement seen in professional sled athletes can lead to lasting cognitive symptoms (Colvin et al., 2009; Talavage et al., 2014; McCradden and Cusimano, 2018). The mechanism by which high frequency sub-concussive impacts can lead to lasting cognitive impairments is poorly understood.

Recently, our lab has shown that mice exposed to a high-frequency of closed head impacts (HFHI) have chronic cognitive impairments (Sloley et al., 2021). The HFHI protocol consists of five closed head impacts given in rapid succession every day for 6 days totaling thirty impacts (Main et al., 2017).

The average college football player receives 21 head impacts per week, with defensive ends receiving 41 head impacts per week (Crisco et al., 2010). To study the physiological changes that occur following head impact, we developed the HFHI mouse model of very mild impact to model the large number of human head impact exposures that occur during a single week of contact sport (Crisco et al., 2010). We recently reported that the HFHI model has very limited TBI or neurodegenerative disease pathology, and does not produce p-tau or Aβ accumulation, and no evidence of inflammation, cell death, or axonal damage outside of the optic tract (Sloley et al., 2021). Using transcriptomics, electrophysiology and pharmacology, we found that the cognitive impairments caused by HFHI are due to impaired long-term potentiation (LTP) in the CA3-CA1 synapse and reduced AMPA/NMDA ratio in CA1 pyramidal cells (Sloley et al., 2021). HFHI mice have strong transcriptomic changes in synaptic signaling and synaptic processes pathways in both the hippocampus and cortex that are present acutely, and are maintained through 1 month post-HFHI (Sloley et al., 2021). The cognitive deficits in HFHI mice can be blocked by pre-administration of memantine, an extrasynaptic NMDA antagonist, supporting the theory that the synaptic adaptations in HFHI mice are driven by short acute bursts of glutamate release at excitatory synapses. Other animal models of mild closed head impacts have demonstrated similar synaptic changes such as shifts in excitatory/inhibitory (E/I) balance in cortical areas (Witkowski et al., 2019). The HFHI model is considerably milder than other published TBI models, including the controlled cortical impact model and lateral fluid percussion models which result in widespread inflammation, cell death and lesion formation. The HFHI model is also pathologically less severe than other published mild TBI models that have associated cognitive deficits with pathologies such as axonal injury, inflammation, and p-tau pathology that are present (Laurer et al., 2001; Prins et al., 2010; Creed et al., 2011; Kane et al., 2012; Ren et al., 2013; Aungst et al., 2014; Mouzon et al., 2014).

Our previous whole cell electrophysiology experiments provide insight into synaptic adaptations on a single cell basis. However, while spatially accurate, they lack information on the integration of the patched neuron into the neuronal network. In contrast LFPs provide rapid measurements from a large population of neurons but have poor spatial localization. Neither of the techniques provide information on the coordinated activity occurring between neurons, known as an ensemble. Neuronal ensembles are understudied in the TBI field, and it is unknown how repetitive head impacts effect neuronal activity at the population level.

In this manuscript, we use Thy1-GCaMP6f transgenic mice expressing fluorescent calcium indicators to measure meso-scale neuronal activity with single cell resolution. While calcium imaging allows for higher spatial specificity, the slow indicator dynamics yield decreased temporal resolution. To observe both the spatial specificity with the temporal resolution, we combine calcium imaging simultaneously with local field potential (LFP) recordings to elucidate microcircuit changes following HFHI using methods recently developed in our lab (Caccavano et al., 2020). Combining these two methods allows for comparison of the relatively slow calcium dynamics in an ensemble specific fashion to canonical and rapid time series data from traditional electrophysiological long-term potentiation (LTP) studies.

## Materials and Methods

### High Frequency Head Impact Model

All procedures were performed in accordance with protocols approved by the Georgetown University Animal Care and Use Committee. Closed head High Frequency Head Impact (HFHI) procedures were performed as previously described (Main et al., 2017; Sloley et al., 2021). Male and female, 2–3-month-old, Thy1-GcAMP6f mice were anesthetized for 3 min in 3% isoflurane in 1.5 L/min oxygen. Mice were placed in the injury device with their unrestrained head resting on a gel pad and isoflurane delivered via a nosecone for an additional minute. The 10 mm diameter Teflon tip was positioned to impact directly on the midline dorsal surface of the head with the front of the impact tip positioned immediately rostral to the eye socket and equidistance from the mouse ears. The area impacted is equivalent to the rostro-caudal length of the parietal bone, with inclusion of rostral areas of the frontal bone. The pneumatically controlled impact was delivered at an impact speed of 2.35 m/s, dwell time of 32 ms, and an impact depth of 7.5 mm. Five impacts were delivered in rapid succession per day for 6 days (totaling 30 hits). Shams received identical handling and anesthesia protocols, but no head impacts.

### Slice Preparation

Acute transverse hippocampal (no preference was given along the dorso-ventral axis) slices were prepared from experimental animals 24 h following the final impact. Brain slices were prepared in NMDG and HEPES-buffered artificial cerebrospinal fluid (aCSF), as previously described (Ting et al., 2014). Briefly, mice were anesthetized in open isoflurane prior to transcardial perfusion, brain dissection, and brain slicing in 0°C NMDG solution (92 mM NMDG, 2.5 mM KCl, 1.25 mM NaH_2_PO_4_⋅2H_2_O, 30 mM NaHCO_3_, 20 mM HEPES, 25 mM glucose, 10 mM sucrose, 5 mM ascorbic acid, 2 mM thiourea, 3 mM sodium pyruvate, 5 mM N-acetyl-L-cysteine, 10 mM MgSO_4_⋅7H_2_O, 0.5 mM CaCl_2_⋅2H_2_O, pH ∼ 7.4, osmolarity ∼ 300–310 mOsm). 350 μm thick transverse slices were prepared using a Vibratome Series 3000. Slices were bisected in the slicing chamber and immediately placed in 32°C NMDG solution for 12 min before being transferred to an incubation chamber containing room temperature carboxygenated HEPES solution (92 mM NaCl, 2.5 mM KCl, 1.25 mM NaH_2_PO_4_2H_2_O, 30 mM NaHCO_3_, 20 mM HEPES, 25 mM glucose, 5 mM ascorbic acid, 2 mM thiourea, 3 mM sodium pyruvate, 5 mM N-acetyl-L-cysteine, 2 mM MgSO_4_⋅7H_2_O, 2 mM CaCl_2_⋅2H_2_O, pH ∼ 7.4, osmolarity ∼ 300–310 mOsm) and were allowed to recover for at least 4 h prior to recording.

### Electrophysiology

Slices were transferred to a Siskiyou PC-H perfusion chamber, anchored to the bottom of the recording chamber and submerged in circulating carboxygenated aCSF (124 mM NaCl, 3.5 mM KCl, 1.2 mM NaH_2_PO_4_⋅2H_2_O, 26 mM NaHCO_3_, 10 mM glucose, 1 mM MgCl_2_⋅6H_2_O, 2 mM CaCl_2_⋅2H_2_O, pH ∼ 7.4, osmolarity ∼ 300–310 mOsm) at 5 mL/min. Recordings were performed with a Multiclamp 700B amplifier (Molecular Devices), digitized to 20 kHz, and low-pass filtered at 2 kHz with a computer running Clampex 11 and DigiData 1440 (Molecular Devices). One recording channel for the LFP was recorded with 0.5–1 MΩ borosilicate pipettes pulled the day of recordings and filled with aCSF and placed in stratum radiatum to measure peak amplitude of field excitatory post synaptic potentials (fEPSPs). A bipolar stimulating electrode was placed in the Schaffer Collaterals. All recording sessions consisted of 10 stimulations given 10 seconds apart. LFP sessions were kept to this short time limit so as not to risk photobleaching of the slice during simultaneous calcium imaging. Recording field potentials between imaging sessions was also not possible since the LFP signal and the calcium imaging signal needed to be time aligned for analysis and we were unable to record multiple separate imaging sessions during a single long LFP recording session. For the input/output response, stimulus pulses ranging from 0 to 90 mA were given to determine the responsiveness of each slice to increasing current pulses. The order of stimulus intensity during input/output sessions was counterbalanced so that the highest intensity stimulus responses weren’t recorded in succession to avoid harming the surrounding tissue or unintentionally inducing a plasticity response (usually depression) in the slice during the input/output response. A stimulus intensity that resembled 30–50% of the maximum response was selected for the high frequency stimulation (HFS) paradigm. Another session was recorded as a baseline before the same stimulus was used in a high-frequency stimulation paradigm to elicit LTP, whereby four tetanic trains were delivered at 100 Hz, 1 s each, with an interstimulus interval of 10 s (Partridge et al., 2000; Sloley et al., 2021). Three LTP sessions were recorded; thirty seconds following HFS, 5 min post HFS, and 10 min post HFS. For LTP analysis, slices that showed depression (<1 normalized peak amplitude) in the post HFS session were excluded from analysis (5/13 sham slices, 4/13 HFHI slices).

### Calcium Imaging

Ca^2+^ ensemble activity of acute slices from Thy1-GcAMP6f mice was simultaneously recorded during LFP sessions with a resonant scanning confocal laser (Thorlabs) at 488 nm. The confocal head is mounted on an Eclipse FN1 microscope (Nikon Instruments). Recordings consisted of seven-hundred and twenty 512 × 512-pixel frames captured at a sample rate of 7.5 Hz using a 40× immersion objective lens covering an area of 350 × 350 μm directly over the stratum pyramidale in the CA1 region. To avoid recording too deep in the slice such that a poor signal/noise ratio or too shallow such that too many calcium loaded cells from slicing were obtained, the z-stepper was used to ensure that each recording took place 30 ± 2 μm below the surface of the slice. Following imaging, imaging files were subject to bleach and motion correction followed by semi-automatic ROI extraction using EZCalcium. LFP and calcium imaging signals were then time matched and feature extraction was then performed (see below).

### Data Analysis

#### Local Field Potential

Local Field Potential preprocessing was done in Clampfit 11 (pClamp, Molecular Devices). Files for the imaging experiments were trimmed around the confocal laser signal for alignment with the calcium transients. Stimulus events were detected using threshold search and stimulus start, peak, and end times were transferred to independent excel files for each session. Cursors were manually placed around the fEPSP response so that slope and maximum response were recorded and transferred to the excel file for that session.

#### Bleach Correction

Raw imaging files were preprocessed as previously described (Caccavano et al., 2020). Briefly, TIF files were converted to change in fluorescence normalized to baseline (ΔF/F) using custom built ImageJ (FIJI) macros. Images were imported using the Bio-Formats plugin and saved as TIF stacks. The resulting stacks were corrected for photo-bleaching using two iterations of the exponential Correct-Bleach plugin for fast and slow bleaching.

#### Motion Correction

Non-rigid motion correction was performed for each session using the EZCalcium package (Cantu et al., 2020). A grid size of 48 × 48 and an upsampling factor of 50 across 200 frames were used for an original template. A sliding bin size of 200 frames with a max shift of 15 pixels was used for subsequent templates for motion correction.

#### ROI Extraction

Somatic ROIs were automatically extracted from motion corrected TIF stacks using the Constrained FOOPSI-SPGL1 algorithm in the EZCalcium package (Cantu et al., 2020). The stacks were spatially downsampled by a factor of two with no temporal downsampling. The algorithm was initialized with a greedy search for an estimated 100 ROIs biased toward an ellipsoid shape. Estimated ROI width was 8 pixels with a merge threshold of 0.9 and a fudge factor of 0.95.

#### ROI Refinement

ROIs were excluded in a semiautomatic fashion using the ROI refinement tool in EZCalcium (Cantu et al., 2020). The automatic exclusion criteria were as follows: no more than 25% baseline drift over the course of 100 frames, baseline stability of 1,000, maximum 2.2 roundness, maximum 3.6 oblongness, area between 5 and 500. Skewness and kurtosis were not assessed at this stage of processing (see below) and zero saturated frames were allowed. A blinded experimenter also manually excluded ROIs on an anatomical basis. Only ROIs in stratum pyramidale were included and ROI’s with largely irregular activity such as those on the border of the field of view or near bright pixels that may have moved into or out of the ROI mask were also excluded.

#### Aligning Local Field Potential With ΔF/F

Calcium events from raw ΔF/F were detected as previously described using custom MATLAB scripts (Caccavano et al., 2020). Briefly, slow changes in fluorescence from further photo-bleaching or drifting of the imaging plane was smoothed using a moving average calculated with locally weighted regression. Baseline corrected ΔF/F traces were then interpolated from the raw frame rate of 7.5 Hz–2 kHz and the LFP signal was downsampled from 20 to 2 kHz for post stimulus analysis. Calcium event detection was set at 4 SD above the calculated baseline with start and end times for events set at 2 SD. Baselines for each ROI was determined by an iterative algorithm of Gaussian fitting to the histogram of all data points. After calculating the baseline mean, SD, and event thresholds, features of detected events for each cell were extracted [start, peak, end, inter-event-interval (IEI), amplitude, and frequency]. To detect events occurring during spontaneous epochs and post stimulation, the interpolated calcium traces were trimmed and aligned with the downsampled LFP trace.

#### Calcium Threshold Normalization

Analysis and visualization of calcium time series data was done using custom MATLAB scripts. To extract ensemble activity, the data set was truncated below the 4 SD event threshold for each cell and normalized to the maximum ΔF/F for that session as previously described (Hamm et al., 2017). Briefly, on a cell-by-cell basis for each session, all values in the time series vector below the 4 SD event threshold were set to 0 and the resulting above threshold signal was divided by the maximum value for that session. This was done for two reasons: (1) calcium events are not binary and thus a relative magnitude should be assessed and (2) spurious pairwise correlations and ensemble peaks resulting from baseline noise are not indicative of true functional activity.

#### Shuffled Dataset

To assess the stimulus evoked ensemble activity, a bootstrapping method was used as previously described (Hamm et al., 2017). For each session, a random number was generated for each ROI to circularly shift the time series signal on a cell-by-cell basis. At each shuffling, the ensemble activity and similarity during the three post-stimulus epochs (see below) was calculated. This step was repeated 1,000 times to generate a dataset with ensemble activity at chance levels.

#### Ensemble Activity

To observe CA1 ensemble activity, the mean for each frame across ROI’s were taken such that the resulting one-dimensional signal represented a percentage of the total active ensemble through time. This upsampled ensemble trace was aligned with the downsampled LFP to determine the periods of stimulus onsets. The continuous ensemble activity was binned into 10 s sweeps of activity between stimulus onsets. For characterization of this activity, sweeps for each session were averaged to give a clear pattern of ensemble activity for each slice and session. Due to the difference in timing of ensemble events (see below) relative to the stimulus, which we attribute to differences in placement of the stimulating electrode and position of the slice along the dorso-ventral axis, the timing of the three epochs of post-stimulus activity characterized in this paper were individually determined for each slice and session using the MATLAB “islocalmax” and “islocalmin” functions for ensemble maxima and minima respectively. ***Ensemble fraction*** during these epochs was calculated by determining the fraction of ROIs with calcium transients during a 10 ms window surrounding the local maxima and minima in the averaged ensemble sweeps for that session.

#### Ensemble Overlap

Overlap of ROI activity during ensemble epochs between stimulations was calculated using a Hamming distance metric between active ROIs of one epoch to the same epoch following a different stimulation. The Jaccard index and cosine similarity metric have been used in the past (Hamm et al., 2017; Caccavano et al., 2020), however, these metrics cannot account for similarity values between two zero matrices, a finding often seen during the ensemble minimum epoch and were not used for this reason.

### Statistics

**FIGURE 1:**
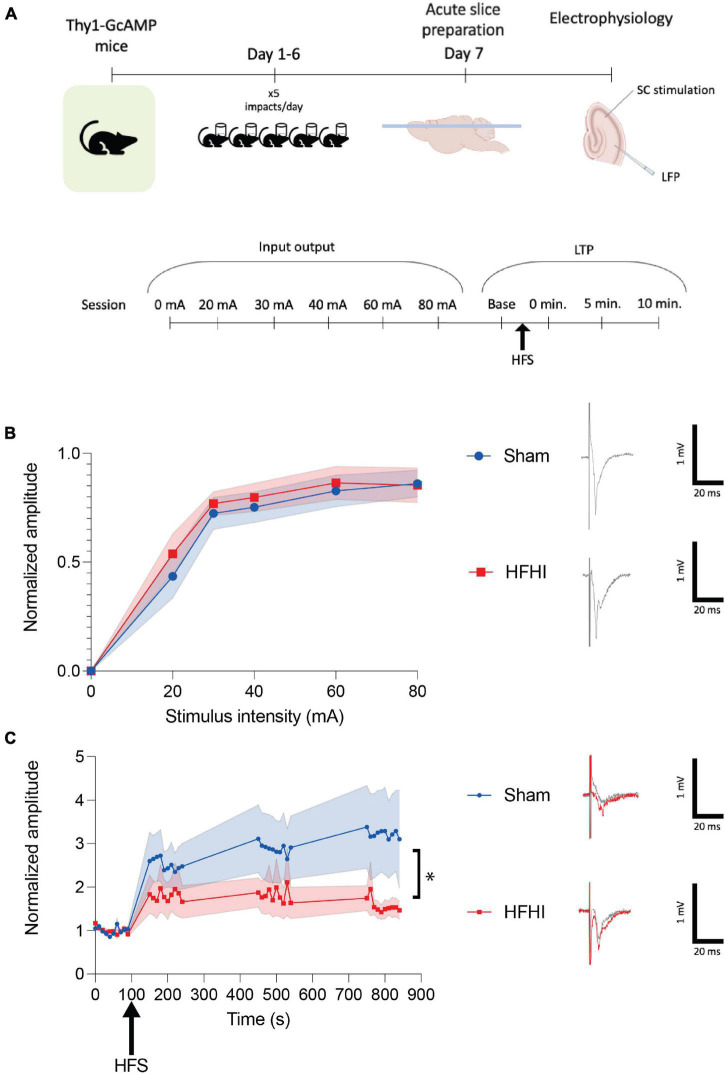
High Frequency Head Impact (HFHI) decreases hippocampal early-LTP. **(A)** Acute slices from Thy1-GCaMP6f mice were prepared 24 h following the last day of HFHI and field recordings in the CA1 field were performed during an abbreviated LTP paradigm. **(B)** HFHI does not alter the input output response (quantified in left, example traces from 80 mA shown on right). **(C)** A significant interaction between time and group in early-LTP was seen following HFHI (quantified in left, example traces from immediately post-HFS shown on right). Shaded regions represent mean ± SEM. Mixed effects analysis, *p < 0.05, *n*_*Sham*_ = 8, *n*_*HFHI*_ = 9.

**FIGURE 2:**
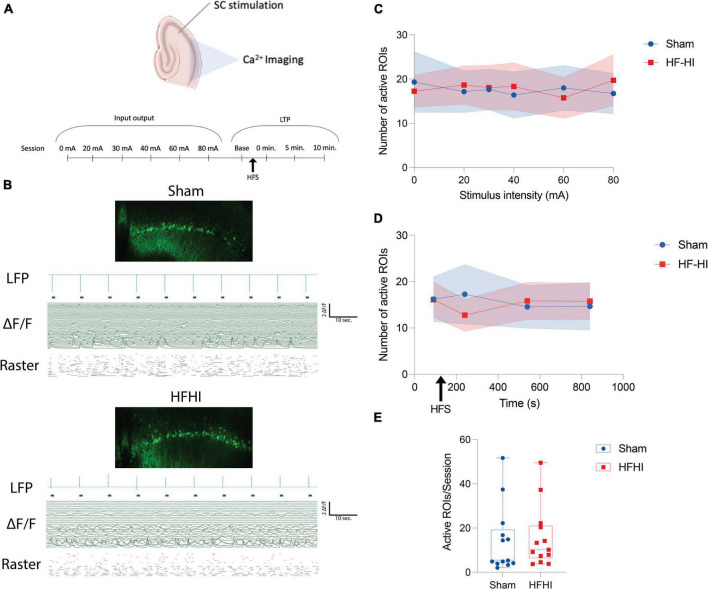
High Frequency Head Impact (HFHI) does not alter number of ROIs with calcium activity. **(A)** Acute slices from Thy1-GcAMP6f mice were prepared 24 h following the last day of HFHI and calcium imaging of the CA1 field was performed during an abbreviated LTP paradigm. **(B)** Example confocal view, aligned LFP, raw calcium traces, and raster plot of sham (top) and HFHI (bottom) slices. **(C)** Semi-automatic ROI extraction revealed no differences in the number of ROIs with activity during the input output sessions, **(D)** LTP sessions, or **(E)** overall number of ROIs extracted per session. Shaded regions represent mean ± SEM in line graphs. *n*_*Sham*_ = 13, *n*_*HFHI*_ = 13.

**FIGURE 3:**
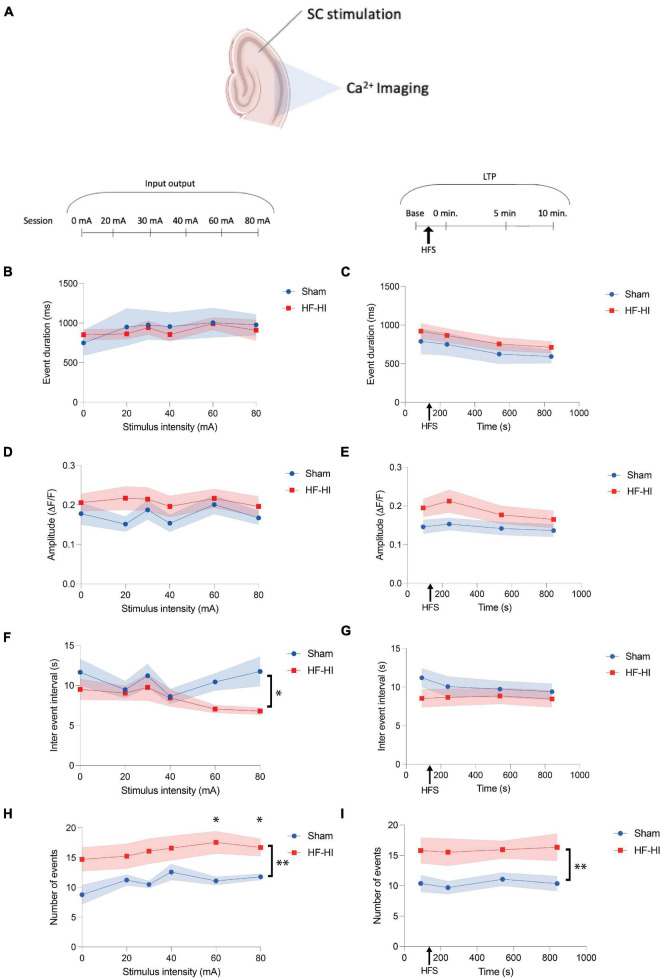
High Frequency Head Impact (HFHI) increases the number of calcium transients but does not alter event features. **(A)** Acute slices from Thy1-GcAMP6f mice were prepared 24 h following the last day of HFHI and calcium imaging of the CA1 field was performed during an abbreviated LTP paradigm. **(B)** HFHI does not alter calcium transient duration during the input output curve or **(C)** following HFS of the Schaffer collaterals. A similar trend was seen for normalized event amplitude in the **(D)** input output sessions and **(E)** LTP sessions. **(F)** HFHI slices displayed a significant decrease in the IEI during the input output sessions, but not **(G)** the LTP sessions. The average number of calcium transients was significantly increased in HFHI slices during **(H)** the input output sessions and **(I)** the LTP sessions. Shaded regions represent mean ± SEM. With brackets: mixed effects analysis, *p < 0.05, ** *p* < 0.01, **(F)** *n*_*Sham*_ = 13, *n*_*HFHI*_ = 13, **(H)** and **(I)** *n*_*Sham*_ = 12, *n*_*HFHI*_ = 13 (one sham outlier removed). Above graph in **(I)** Šídák’s multiple comparisons test, *p < 0.05, *n*_*Sham*_ = 12, *n*_*HFHI*_ = 13.

All statistics were done in Prism 8 (GraphPad). In total, 13 slices from 8 sham animals (5 animals with two slices) and 13 slices from 9 HFHI animals (4 animals with two slices) were recorded from. “n” values in figure legends refer to the number of slices. The n number can vary from analysis to analysis as some sessions were excluded due to slice shifting or a diminishing number of ROIs below the determined minimum of 3 during one session but valid during other sessions. Outliers were defined as those slices showing a value 1.5 times the interquartile range above or below either quartile. Due to this, a mixed effects analysis with Šídák’s *post hoc* comparisons were performed on the LFP response and continuous calcium metrics (Figures 1B,C, 2C,D, 3B–I, 5A–F, 6A–F) throughout the paradigm. Shaded regions on line graphs indicate mean ± SEM.

**FIGURE 4:**
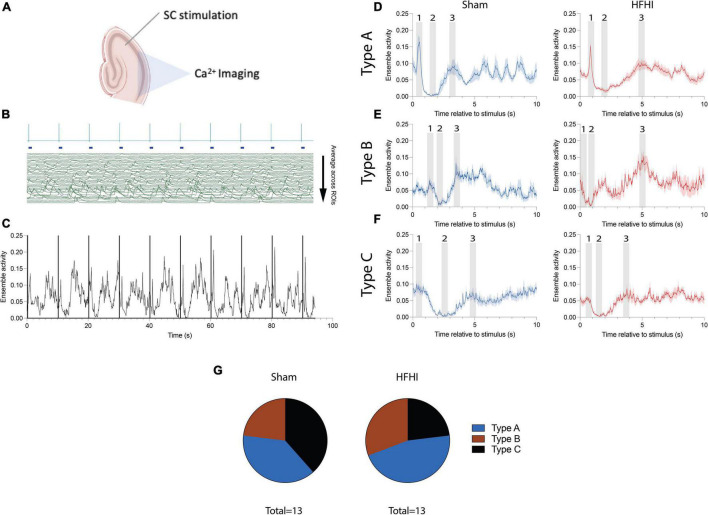
High Frequency Head Impact (HFHI) and sham slices both display distinct ensemble activity following SC stimulation. **(A)** Acute slices from Thy1-GCaMP6f mice were prepared 24 h following the last day of HFHI and calcium imaging was performed simultaneously with LFP recordings of the CA1 field during an abbreviated LTP paradigm. **(B)** Example HFHI LFP (top, blue) aligned with raw calcium traces (bottom, green) from one recording. Ensemble activity was obtained by setting individual ROI calcium activity below threshold (4 SD above the baseline) to zero and averaging across all ROIs at each timepoint. **(C)** Example ensemble activity (black) with stimulus onset overlain (purple) for one session. Calcium ensemble activity following stimulus onset was visualized by binning ensemble activity into 10 s sweeps between stimulus onsets and averaging all the sweeps. We characterized various patterns of post-stimulus ensemble activity generally consisting of **(D)** a prominent maximum shortly <1 s after the stimulus (epoch 1), an ensemble minimum 1–3 s following stimulation (epoch 2), and a second ensemble maximum following the minimum (epoch 3) which was usually less prominent although slices from both groups displayed **(E)** more prominent second maxima or **(F)** only a transient ensemble inactivation with no maxima. **(G)** Pie charts showing distribution of patterns classified into Type A (1st maximum and ensemble minimum), Type B (2nd maximum an ensemble minimum), and Type C (ensemble minimum only). Shaded regions represent mean ± SEM across 9 sweeps in line graphs.

**FIGURE 5:**
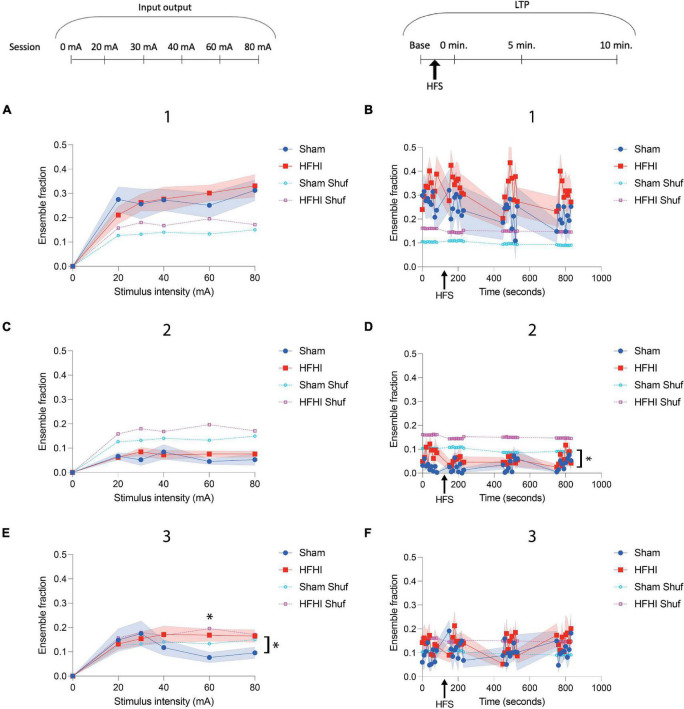
High Frequency Head Impact (HFHI) increases ensemble size during coordinated activity periods. **(A)** HFHI and sham slices display increasing number of events during the first ensemble maximum with increasing stimulus intensity, but no differences were observed between the group. **(B)** Potentiation of the CA1 field with HFS did not alter transient frequency during the first ensemble maximum. **(C)** Calcium transient frequency during the ensemble minimum remained low for both groups throughout the input/output paradigm relative to the ensemble maxima and did not differ with stimulus intensity. **(D)** A significant difference in the fraction of ensemble with calcium transients during the ensemble minima between the groups was seen **(E)** Sham ensemble activity during the second ensemble maxima appeared to be inversely related with stimulus intensity while HFHI slices remained stable at higher stimulus intensities, leading to a significant interaction between time and group. **(F)** The activity during the second ensemble maximum was not altered by HFS and did not differ between groups. Shaded regions represent mean ± SEM. With brackets: mixed effects analysis, *p < 0.05, comparing real data (non-shuffled) from each group. Above graph in E) Šídák’s multiple comparisons test, *p = 0.05 *n*_*Sham*_ = 13, *n*_*HFHI*_ = 13.

**FIGURE 6:**
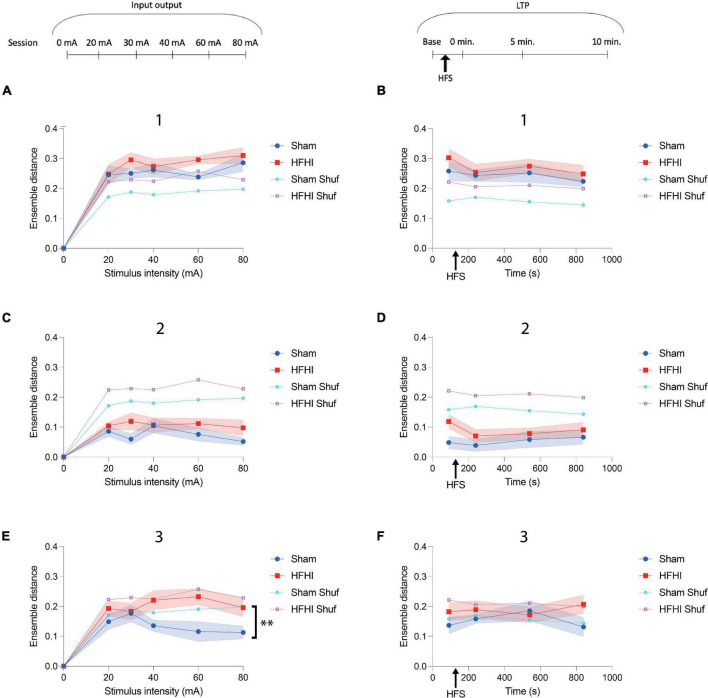
High Frequency Head Impact (HFHI) increases post stimulus ensemble distance during second maximum. **(A)** HFHI slices displayed significantly increased ensemble distance during the first maximum during the 60 mA input output session, despite the mixed effects analysis not significantly differing between groups or **(B)** during the LTP sessions. **(C)** Ensemble distance during the minimum time range did not differ significantly between groups in the input output session, however, **(D)** during the LTP sessions HFHI displayed a trend toward increased ensemble distance. **(E)** During the second maximum, HFHI slices displayed significantly increased ensemble distance, especially at higher stimulus intensities, but **(F)** not during the LTP sessions. Mixed effects analysis, **p < 0.01, comparing real data (non-shuffled) from each group, *n*_*Sham*_ = 13, *n*_*HFHI*_ = 13.

### Code Availability

MATLAB code for semiautomatic extraction of calcium ROIs using EZCalcium is openly accessible (Cantu et al., 2020). MATLAB functions for aligning raw calcium traces with LFP and extracting calcium events from Caccavano et al. (2020) can be found here: https://github.com/acaccavano/SWR-Analysis. Custom MATLAB software used in the present study for feature extraction and ensemble analysis can be found here: https://github.com/dpchapma/CalciumImagingAnalysis.

## Results

### High-Frequency Head Impact Impairs Early-LTP 24 h Post-injury

To assess synaptic plasticity 24 h following HFHI, we investigated early-LTP in the CA3-CA1 circuit (Figure 1A). Mixed effects analysis revealed no changes to the input-output response in HFHI brain compared to sham brain (Figure 1B). Following 10 baseline stimulations at a stimulus intensity that was 30–50% of the maximum response a high frequency stimulation (HFS) was given (ending at time 0) and the relative field response was measured 0-, 5-, and 10-min post HFS (Figure 1C). We found a significant interaction between injury group and time following HFS despite between group effects not reaching significance [mixed effects analysis: Time × Group, *p* = 0.0104, *F*(39, 553) = 1.633; Group, *p* = 0.1291, *F*(1, 15) = 2.580; Time, *p* = 0.0061, *F*(2.073, 29.40) = 5.987; *n*_*Sham*_ = 8, *n*_*HFHI*_ = 9]. These results corroborate previous work from our lab showing a reduction in early-LTP in the absence of a change in the input/output curve (Sloley et al., 2021).

### High Frequency Head Impact Does Not Change Number of Active ROIs

To understand the inability of the repeat head impact brain to fully potentiate, we examined calcium dynamics in the CA1 field during the early-LTP paradigm. The active constituents of the CA1 ensemble were assessed during the early-LTP paradigm by imaging the CA1 field of Thy1-GCaMP6f animals simultaneously during the CA3-CA1 LTP protocol (Figure 2A). To assess the number of active neurons in the CA1 field following HFHI, somatic ROIs were extracted, and raw calcium traces were aligned with the LFP signal (see section “Materials and Methods”) in both groups (Figure 2B). Calcium transients were defined as periods of time in which the ΔF/F reached 4 SD above the baseline for each ROI. The number of ROIs with calcium transients did not differ between the groups during the input/output sessions (Figure 2C) or the LTP sessions (Figure 2D). We also quantified the number of ROIs with calcium transients across all sessions for each slice and found no difference between the groups (Figure 2E). This corroborates previous reports from our group that HFHI does not lead to neuron cell death. However, due to the importance of calcium following TBI, we next quantified differences in calcium transients following HFHI.

### Number of Calcium Transients Are Increased in High Frequency Head Impact Slices

We next sought to understand whether the calcium event dynamics differed between groups across the stimulation paradigm. Average transient amplitude, duration, IEI, and number of transients were averaged across ROIs for each input output sessions and an abbreviated LTP paradigm (Figure 3A). Only active ROIs were considered and all events across the sessions were considered irrespective of each transient time relation to the stimulus onset. Event duration did not differ between the groups during the input output sessions (Figure 3B) and LTP sessions (Figure 3C). Similarly, calcium transient ΔF/F remained unchanged between the groups during both input output sessions (Figure 3D) and LTP sessions (Figure 3E). Calcium transient IEI displayed an inverse relationship with stimulus intensity in HFHI slices, but no relationship with sham slices, resulting in a significant decrease of IEI in HFHI across the input output sessions [Figure 3F, Mixed effects analysis: Stimulus × Group, *p* = 0.2260, *F*(5, 102) = 1.4; Group, *p* = 0.0313, *F*(1, 24) = 5.234; Stimulus, *p* = 0.3399, *F*(2.660, 54.27) = 1.134; *n*_*Sham*_ = 13, *n*_*HFHI*_ = 13]. This change in IEI, however, was not reflected during the LTP sessions (Figure 3G) which took place at relatively low stimulus intensities (∼20–50 mA). The number of calcium transients per ROI were significantly increased in HFHI slices during the input/output sessions [Figure 3H, Mixed effects analysis: Stimulus × Group, *p* = 0.4038, *F*(1, 23) = 9.31; Group, *p* = 0.0057, *F*(1, 23) = 9.31; Stimulus, *p* = 0.1498, *F*(2.706, 52.50) = 1.879; *n*_*Sham*_ = 11, *n*_*HFHI*_ = 12; Šídák’s multiple comparisons test; 60 mA, *p* = 0.0326; 80 mA *p* = 0.0447] as well as during the LTP sessions [Figure 3I, Mixed effects analysis: Time × Group, *p* = 0.9892, *F*(3, 49) = 0.03; Group, *p* = 0.0092, *F*(1, 21) = 8.241; Time, *p* = 0.8361, *F*(2.568, 41.94) = 0.2439; *n*_*Sham*_ = 11, *n*_*HFHI*_ = 12]. These data demonstrate that individual ROIs of HFHI slices differ in the number of calcium transients, but not amplitude or duration.

### High Frequency Head Impact and Sham Slices Show Coordinated Patterns of Post Stimulus Ensemble Activity

We next sought to understand how individual calcium ROIs were functionally connected during the early-LTP paradigm. Calcium imaging offers the ability to record from tens-of-thousands of neurons simultaneously to assess network changes *in vivo* (Cossart et al., 2003; Hamm et al., 2017). Ensembles are defined as groups of transiently coactive neurons and are thought to be one of the most basic units of neural computation (Buzsaki, 2010). We assessed the correlation between individual pairs of cells but found no significant relationships or differences between groups (Mean ± SEM pairwise correlations across all sessions: Sham = 0.1177 ± 0.015, HFHI = 0.1294 ± 0.010). To assess CA1 ensemble activity following HFHI, collective calcium activity was mapped across our stimulation paradigm (Figure 4A). Ensemble activity was calculated by averaging the normalized activity across ROIs (Figure 4B: example session with individual ROIs). The resultant trace (Figure 4C), which reflects the averaged ensemble activity across time, was time aligned with the downsampled LFP signal (Figure 4C: vertical lines) to assess ensemble activity in the seconds following each stimulus. We then binned the ensemble activity for each recording into 10-second inter-stimulus sweeps (9 total) and averaged ensemble activity across each sweep. Both sham and HFHI slices showed coordinated patterns of activity and we observed three distinct features (Figures 4D–F) numerically illustrated in the shaded regions as (1) first ensemble maximum, (2) ensemble minimum, and (3) second ensemble maximum. All three features are best illustrated from example sessions in Figure 4D where the first maximum (epoch 1) which usually occurred <1 s followed by the ensemble minimum (epoch 2) occurring 1–3 s following the stimulus and ending with the second ensemble maximum (epoch 3) occurring within 5 s of the stimulus. Both sham and HFHI slices displayed patterns of activity with one or all three of these patterns. Interestingly, across groups as well as individual slice ensemble patterns, the ensemble minimum (epoch 2) was the most consistent pattern of (in)activation seen and was observed in every slice in both groups at the highest stimulus intensity.

We classified slices as either having a Type A (1st maximum is greater and an ensemble inactivation, Example 4D), Type B (2nd maximum and no 1st maximum Example 4E), or Type C (no maxima, only a transient inactivation of the ensemble, Example 4F) patterns at the highest stimulus intensity recorded for that slice (Figure 4G). We chose the highest stimulus intensity for this classification since the consistency and magnitude of these patterns at lower stimulus intensities was reduced compared to higher intensities. Despite this fact, slices which displayed a particular ensemble activity pattern at higher intensities displayed the same pattern at lower intensities as well, albeit with diminished consistency from stimulus to stimulus. Chi-squared analysis revealed no difference between the prevalence of these patterns amongst slices. The timing of each feature did not differ between groups or with stimulus intensity during the input/output sessions and time during the LTP session. These traces demonstrate a novel pattern of CA1 calcium ensemble activity following SC stimulation and show that these patterns are qualitatively unaffected by HFHI.

### High Frequency Head Impact Increases CA1 Ensemble Fraction During Coordinated Activity Periods

We next sought to quantify ROI activity during the ensemble epochs in sham and HFHI slices to characterize differences in these activity patterns in the repeat head impact brain. To quantitatively assess ensemble activity following stimulus onset, we calculated the fraction of ROIs with calcium transients during 10 ms windows in the three major events described in Figure 4 were calculated. The activity of the raw ensemble was compared to a shuffled dataset generated similar to Hamm et al. (2017). During the input/output sessions, a positive correlation was seen between stimulus intensity and the ensemble fraction during the first ensemble maximum (Figures 4D–F, epoch 1). Ensembles from both groups displayed a larger fraction of active ROIs compared to their shuffled datasets, however, there was no difference observed between groups (Figure 5A). ROI fraction during the first ensemble maximum activity was not altered by HFS and did not differ between groups but remained well above the fraction seen in the shuffled datasets (Figure 5B). Unsurprisingly, the ensemble fraction during the ensemble minimum (Figures 4D–F, epoch 2) was substantially lower than that of the first ensemble maximum and the shuffled data, however it did not differ between groups or correlate to stimulus intensity (Figure 5C). During the LTP sessions, a significant difference between groups was seen in the ensemble fraction during the ensemble minimum with HFHI slices displaying elevated activity during this epoch compared to sham slices [Figure 5D, Mixed effects analysis: Time × Group, *p* = 0.7627, *F*(35, 644) = 0.8189; Group, *p* = 0.0462, *F*(1, 22) = 4.465; Time, *p* = 0.3541, *F*(6.016, 110.7) = 1.122; *n*_*Sham*_ = 12, *n*_*HFHI*_ = 12]. Lastly, assessing the second ensemble maximum (Figures 4D–F, epoch 3) revealed an inverse relationship between stimulus intensity and ensemble fraction in sham slices, with the ensemble fraction dropping below chance levels with increasing stimulus intensity. This did not occur in HFHI slices, which remained at chance levels throughout the paradigm. This resulted in a significant interaction between time and group with HFHI slices displaying an increased ensemble fraction [Figure 5E, Mixed effects analysis: Stimulus × Group, *F*(5, 85) = 2.398; Group, *p* = 0.3107, *F*(1, 24) = 1.072; Time, *p* = 0.6682, *F*(2.882, 59.81) = 0.5122; Šídák’s multiple comparisons test; 60 mA, *p* = 0.05; *n*_*Sham*_ = 13, *n*_*HFHI*_ = 13]. However, there was no difference between groups during the second ensemble maximum throughout the LTP sessions, which took place at relatively low stimulus intensities (∼20–50 mA), nor did either group differ from their shuffled dataset (Figure 5F). We generally found an increase in number of calcium transients in HFHI slices compared to shams when assessing transients across the entire session (Figures 3F–I) and revealed a similar trend in the ensemble analysis during the second ensemble maximum (Figure 5E) and ensemble minimum (Figure 5D) during the input/output curve and LTP sessions, respectively. These findings illustrate an abnormality in HFHI animals in the normal calcium transient firing during specific time periods related to stimulus induced ensemble activity.

### Repetitive Ensemble Firing Is Partially Diminished Following High Frequency Head Impact

Previous reports of calcium activity from various brain regions *in vivo* and *in vitro* have revealed that ensemble activations frequently occur through a similar set of ROIs firing in a repeating fashion (Cossart et al., 2003; Villette et al., 2015; Hamm et al., 2017) and plasticity events are known to alter the makeup of the ensemble *in vitro* (Yuan et al., 2011). To assess the repetitive nature of specific ROIs during the novel post-stimulus ensemble epochs described in the present study (Figures 4D–F), we quantified a distance metric, a comparison between the pattern of ROIs that fired or didn’t fire, following each stimulation and compared them against a shuffled dataset for reference (Figure 6). Generally, groups did not differ in ROI set distance during the first ensemble maximum (1) during either the input/output (Figure 6A) or LTP sessions (Figure 6B). Interestingly, there was no difference in distance in the ensemble minimum epoch (2) between group, stimulus intensity during the input output sessions (Figure 6C), or time during the LTP sessions (Figure 6D), however, ensemble distance was well below that of the shuffled datasets during this epoch in both groups. Contrarily, we observed a significant increase in HFHI ensemble distance during the second ensemble maximum (3) of the input/output sessions [Figure 6E, Mixed effects analysis: Stimulus × Group, *p* = 0.1388, *F*(4, 83) = 1.78; Group, *p* = 0.0091, *F*(1, 24) = 8.334; Stimulus, *p* = 0.8951, *F*(3.401, 70.58) = 0.2319; *n*_*Sham*_ = 13, *n*_*HFHI*_ = 13]. Like our findings on ensemble fraction during this epoch (Figure 5E), we observed an inverse correlation between ensemble distance and stimulus intensity in sham mice, but not HFHI, despite starting at similar levels to both the HFHI group and shuffled datasets at the lower intensities (Figures 5E, 6E). No difference was seen between group, time, or against the shuffled datasets, however, during the LTP sessions (Figure 6F). Collectively, this data shows an abnormality in repetitive ROI activation during patterns of ensemble activity following HFHI.

## Discussion

Sub-concussive impacts or sustained high frequency low amplitude cranial movement seen in high school football players, soccer players, and professional sled athletes can lead to lasting cognitive symptoms (Colvin et al., 2009; Talavage et al., 2014; McCradden and Cusimano, 2018); however, the mechanism by which these impacts can contribute to changes in brain function is poorly understood. Here we used field recordings to demonstrate that Thy1-GCaMP6f mice exposed to HFHI have reduced early-LTP, and matched calcium imaging to demonstrate that CA1 neurons have increased calcium activity and impaired ensemble dynamics.

### High Frequency Head Impact Impairs Early-LTP and Increases Calcium Activity

Impaired hippocampal plasticity has been reported in several TBI models of various severity and frequency (Miyazaki et al., 1992; Albensi et al., 2000; Goldstein et al., 2012; Aungst et al., 2014; Mei et al., 2018; Tagge et al., 2018). However, the heterogenous pathologies that occur following TBI, such as neuron death, axon shearing, reduction in synapses, and synaptic adaptation can all affect plasticity. For this reason, the mechanism behind impaired LTP in other TBI models can often be difficult to dissect out due to the multi-pathology phenotypes. Contrarily, we do not observe hippocampal cell death or inflammation in the HFHI model, but instead reported isolated synaptic changes that underlies this altered plasticity (Sloley et al., 2021). In the present study we extended those results to Thy1-GCaMP6f mice exposed to sham or HFHI and found that HFHI reduced hippocampal early-LTP which occurs without measurable change in the input/output curve, similar to our previous findings in HFHI C57Bl/6 mice (Sloley et al., 2021).

We hypothesized that synaptic adaptations occur following HFHI, whereby the neurons adapt to the head impacts in a manner that is designed to protect the brain against future insult. If true, we would expect that the synaptic adaptations would act to alter the downstream events occurring in response to TBI-induced glutamate release, including changes to calcium dynamics. In this study, we report that HFHI slices have similar numbers of somatic ROIs to sham animals but show increased calcium transient frequency in these ROIs. These data contrast with previous calcium imaging done *in vivo* hyperacutely (∼1 h) following two-successive days of blast injury, which showed hypoactivity, including vastly decreased intracellular calcium activity and altered calcium event dynamics (Hansen et al., 2018). There are several experimental differences that could account for these contrasting results. The blast model is more severe, has a lower frequency of impacts, and imaging was performed at a much earlier timepoint relative to the injury when compared to our study (Hansen et al., 2018). Furthermore, the recent characterization of HFHI by our group showed a general pattern of decreased excitability in whole-cell patch clamp recordings of HFHI neurons (Sloley et al., 2021). Despite this hypoexcitability phenotype in whole cell configuration, it is possible to see increased calcium dynamics possibly originating from several sources: increased spontaneous glutamate release, decreased reuptake, disinhibition, and decreased intracellular calcium sequestration. In a single and repetitive controlled cortical impact model, a much more severe model of TBI, single cell electrophysiology and calcium imaging revealed increased baseline calcium accompanied by hyperexcitability in the input/output response in TBI groups (McDaid et al., 2021). This both corroborates and contrasts our results as we see an increase in calcium transients but no change in the input/output response in HFHI animals. Further work using pharmacological isolation is needed to understand the source of increased calcium transients seen in the present study.

### The CA1 Field Shows Coordinated Patterns of Ensemble Activity Following Schaffer-Collateral Stimulation

Ensemble dynamics *in vitro* are impossible to reasonably correlate to behavior states; however, they can provide useful insight into network integrity at the meso- and micro-circuit level. In our experiment, we measured ensemble activations following stimulation by averaging across ROI activity and aligning this activity trace with the stimulus onsets. This analysis revealed a pattern of activity similar to a previous report using genetically encoded voltage indicators (GEVI) *in vitro* (Nakajima et al., 2021) but, to our knowledge, has not been previously characterized using Thy1-GCaMP6f animals. We broke the activity down into three distinct features that occurred between ∼0 and ∼5 s following stimulus onset and consisted of (1) a first ensemble maximum; a narrow (∼0.2 s) and prominent peak usually between 0 and 1 s following stimulus onset. (2) An ensemble minimum; a significant and consistent decrease in ensemble activity, often times even to 0% of the ROIs being active and lasting around 1 s. We attribute this to lateral inhibition in the CA1 field following excitation of somata in the first maximum. This hypothesis is supported by the previous study of CA1 stimulation using GEVI mice as the similar dip in activity following stimulation was sensitive to GABA blockers (Nakajima et al., 2021). This decrease in ensemble activity was the most consistent across slices, groups, and sessions. (3) A second ensemble maximum; a broader peak than the first ensemble maximum that usually was of less amplitude. This third epoch is the most variable in terms of timing and amplitude of the three epochs identified.

By combining these three features (see above) we observed one of the three patterns (type A, B, or C) in all slices and the type of pattern observed did not change across different stimulus intensities within the same slice, although some slices did not display coordinated ensemble activity at the lowest stimulus intensity of the input output curve. Some animals had multiple slices recorded from (Sham, *n* = 5; HFHI, *n* = 4 animals with two slices), some of which displayed different patterns of activation between slices of the same animal. This precludes the possibility that the type of coordinated ensemble activity observed is implicit to the animal but rather reflects slight differences in experimental parameters at the time of recording. Thus, we attribute the qualitative difference in coordinated activity patterns (Type A, B, or C) to slice level along the dorso-ventral axis and/or the exact placement of the stimulating electrode relative to the CA1 field.

While our pattern of ensemble activity reflects a mirror image of that seen reported from Nakajima et al., their report drew data from all regions of the imaging field rather than just looking at active soma in stratum pyramidale and did not look at plasticity inducing stimulations. Furthermore, the timescale for activity in the present study is significantly drawn out due to the slow indicator dynamics of GCaMP6f when compared to GEVIs. Despite these differences, ensemble activity extracted from active soma in the CA1 from Thy1-GCaMP6f mice and mined activity using GEVIs showed similar qualitative properties following stimulation. Other groups have reported similar calcium ensemble spikes in the dorsal CA1 (Xia et al., 2017) and amygdala (Corder et al., 2019) *in vivo* relating to conditional behavior paradigms as opposed to stimulation paradigms, however, none have reported the same pattern of lateral inhibition following stimulus onset.

Qualitatively, no difference was seen between sham and HFHI slices in the pattern of post-stimulus ensemble activity. These findings along with the unchanged input/output curve during the field recordings would indicate a grossly intact network architecture in the CA1 field, once again reaffirming our findings that neuron death, axon shearing, or widespread inflammation are unlikely causes of decreased learning and plasticity in HFHI.

### High Frequency Head Impact Alters ROI Activation During Periods of Coordinated Ensemble Activity

We quantified the percentage of ROIs with calcium transients (ensemble fraction) during the periods described above for both the input/output and LTP sessions and compared groups against each other and a shuffled dataset. Ensemble activity from both groups during the first maximum and ensemble minimum showed an increase and decrease in ensemble activity compared to the shuffled data, respectively. During the second maximum, only the sham slices significantly deviated from the shuffled datasets at higher stimulus intensities during the input/output sessions, but not the LTP sessions, which took place at lower stimulus intensities (∼20–40 mA). When comparing the real data from both groups, we found a significant increase in HFHI slices of ensemble fraction during the ensemble minimum of the LTP sessions. This corresponds to the decreased IEI seen in HFHI slices during the input/output sessions, which was only observed at the two highest stimulus intensities, indicating that the general decrease in IEI at higher stimulus intensities can be accounted for by events occurring during the ensemble minimum.

Some authors hypothesize that white matter disruptions or neuronal degeneration following rmTBI are the cause of network disruptions (Sharp et al., 2014; Wolf and Koch, 2016; Schumm et al., 2020). However, other work done *in vivo* following a fluid percussion injury showed decreased CA1 network synchrony despite no change in firing rates or neuron cell death (Koch et al., 2020) and further *in vitro* work suggests changes to NMDA subunit density and distribution due to mechanical forces reduces network correlation (Patel et al., 2014). This is complemented by modeling work showing that altered NMDA receptor properties following TBI showed deficient spike time dependent plasticity and computational properties in injured networks compared to uninjured ones without the removal of network components (Gabrieli et al., 2021). Thus, we attribute the differences seen in ensemble fraction activity across the stimulation paradigm in HFHI to a synaptic mechanism.

There are several possible mechanisms that could explain this finding. Given that the ensemble minimum is likely due to lateral inhibition in the CA1 field (Nakajima et al., 2021), one possible mechanism to explain the observed increase in the HFHI slices is a disruption in the microcircuit between pyramidal cells and inhibitory interneurons. Given that qualitative pattern of coordinated ensemble activity can differ across slices of the same animal, but not across stimulus intensity within the same slice (see above), we believe that efficacy of lateral inhibition in the CA1 field of HFHI slices may be compromised and lead to the altered ensemble fractions seen in this study. This is in line with previous work done in a more severe model of TBI which found prominent increases in cortical E/I balance in the hours following injury, but these effects were dampened by 48 h post injury (Witkowski et al., 2019). Similarly, another report found decreased cortical inhibition in a fluid percussion model, however, this inhibition occurred at later timepoints (Hsieh et al., 2017). Changes to inhibitory neurons following HFHI have yet to be characterized, however, their prominent role in hippocampal ensemble coordination during network events such as sharp-wave ripples (Buzsaki, 1986) suggest that changes to inhibition in the CA1 microcircuit could contribute to the decreased ensemble coordination observed in the present study.

A second possible explanation is decreased glutamate reuptake at the CA3-CA1 synapse. A growing body of literature in the Alzheimer’s field has pinpointed impaired glutamate reuptake as a possible source of hyperactivity seen in various models of pathology (Busche et al., 2008; Zott et al., 2019). While these studies showed hyperactivity *in vivo* and not naturally *in vitro*, a similar mechanism could explain both the increased ensemble activity during minima and rebound maxima following stimulation as well as the diminished LTP from field recordings. Lingering glutamate in the synaptic cleft can activate extrasynaptic NMDA receptors, leading to a slower and more drawn-out entry of calcium into cells, a pattern most often associated with synaptic depression. If this were true in the present study, we may have expected to see an increase in event duration. While we did not see this effect, it is possible that an extension of the event duration is present closer to the synapse at dendritic ROIs but is washed out by the time transients reach the soma.

Lastly, calcium indicators are proxy for neural activity, however, in theory not all calcium events represent neural activity. Thus, a third explanation for the general hyperactivity in both individual calcium transients and coordinated ensemble activity in HFHI slices could be due to decreased intracellular calcium sequestration mechanisms. Previous studies in a single and repetitive CCI model showed increased levels of basal calcium due to voltage-gated calcium channels but found that ryanodine receptor (RYR) calcium responses decreased with repetitive head impacts (McDaid et al., 2021), indicating that intracellular calcium is not the source of increased calcium events we see. Our HFHI model is notably less severe than a CCI model of TBI, however, further work to pharmacologically block the intracellular RYR is necessary to understand this mechanism.

While the exact mechanism of ensemble asynchrony in the present study is currently unknown, our characterization of the HFHI model showed a persistent decrease in the AMPA/NMDA ratio as well as changes in neuronal excitability 24 h following HFHI despite no changes in input/output responses or hippocampal neuron damage (Sloley et al., 2021), indicating that the altered ensemble patterns seen in the present study are not likely due to white matter disruptions or neuron cell death, but rather alterations to excitatory and/or inhibitory synaptic function.

## Conclusion and Limitations

The results of this experiment show that HFHI alters CA1 calcium ensemble dynamics and provide further evidence of physiological adaptations in neurons following non-damaging head impacts. Decreased, but not fully abolished, plasticity responses and functional changes to activity in neurons are likely to contribute to behavioral deficits seen in the repeat head impact brain. The present study employed calcium imaging simultaneously with LFP recordings in the CA1 field during a plasticity paradigm. While these techniques offer the ability to simultaneously image dozens of neurons, we chose only to select somatic ROIs in stratum pyramidale. This was initially done from an empirical perspective as it is rarer to find slices that contain dendritic ROIs compared to somatic ROIs. This analysis shed light on stimulus evoked ensemble activity, however, limiting ROI extraction to soma only could potentially limit the ability of our experimental design to capture plasticity induced calcium events that may be occurring if dendritic ROIs in stratum radiatum were assessed. As such, the potentiation of the slices had little effect on the somatic calcium activity (spontaneous and stimulus evoked) observed in this study as somatic analysis only represents an integration of upstream synaptic inputs and not a direct measure of synaptic activity. Future work, likely involving pharmacological isolation, is needed to parse apart the exact mechanisms of stimulus evoked ensemble activity to determine the role of lateral inhibition and glutamate reuptake in the seconds following SC stimulation. A further limitation of this study is the inclusion of only the first ∼10 min. of the LTP response. Early-LTP is reflective of some, but not all mechanisms of plasticity in CA1 pyramidal cells and the study would be strengthened by extending the field recordings up to an hour post-tetanus. This was not done due to the large data requirements and possible photobleaching that comes with calcium imaging (see section “Materials and Methods”).

## Data Availability Statement

The raw data supporting the conclusions of this article will be made available by the authors, without undue reservation.

## Ethics Statement

The animal study was reviewed and approved by the Georgetown University Animal Care and Use Committee.

## Author Contributions

DC, SS, AC, SV, and MB conceptualized the project and contributed to the experimental design. SS and DC performed the experiments and collected the data. DC and AC wrote the code to analyze the data. DC performed the statistical analysis, wrote the manuscript, and created the figures. All authors revised, edited, and approved the submitted version of the manuscript.

## Conflict of Interest

The authors declare that the research was conducted in the absence of any commercial or financial relationships that could be construed as a potential conflict of interest.

## Publisher’s Note

All claims expressed in this article are solely those of the authors and do not necessarily represent those of their affiliated organizations, or those of the publisher, the editors and the reviewers. Any product that may be evaluated in this article, or claim that may be made by its manufacturer, is not guaranteed or endorsed by the publisher.

## References

[B1] Albensi B. C. Sullivan P. G. Thompson M. B. Scheff S. W. Mattson M. P. (2000). Cyclosporin ameliorates traumatic brain-injury-induced alterations of hippocampal synaptic plasticity. *Exp. Neurol.* 162 385–389. 10.1006/exnr.1999.7338 10739643

[B2] Aungst S. L. Kabadi S. V. Thompson S. M. Stoica B. A. Faden A. I. (2014). Repeated mild traumatic brain injury causes chronic neuroinflammation, changes in hippocampal synaptic plasticity, and associated cognitive deficits. *J. Cereb. Blood Flow Metab.* 34 1223–1232. 10.1038/jcbfm.2014.75 24756076 PMC4083389

[B3] Blennow K. Brody D. L. Kochanek P. M. Levin H. Mckee A. Ribbers G. M. (2016). Traumatic brain injuries. *Nat. Rev. Dis. Primers* 2:16084. 27853132 10.1038/nrdp.2016.84

[B4] Busche M. A. Eichhoff G. Adelsberger H. Abramowski D. Wiederhold K. H. Haass C. (2008). Clusters of hyperactive neurons near amyloid plaques in a mouse model of Alzheimer’s disease. *Science* 321 1686–1689. 10.1126/science.1162844 18802001

[B5] Buzsaki G. (1986). Hippocampal sharp waves: their origin and significance. *Brain Res.* 398 242–252. 10.1016/0006-8993(86)91483-6 3026567

[B6] Buzsaki G. (2010). Neural syntax: cell assemblies, synapsembles, and readers. *Neuron* 68 362–385. 10.1016/j.neuron.2010.09.023 21040841 PMC3005627

[B7] Caccavano A. Bozzelli P. L. Forcelli P. A. Pak D. T. S. Wu J. Y. Conant K. (2020). Inhibitory parvalbumin basket cell activity is selectively reduced during hippocampal sharp wave ripples in a mouse model of familial Alzheimer’s disease. *J. Neurosci.* 40 5116–5136. 10.1523/JNEUROSCI.0425-20.2020 32439703 PMC7314414

[B8] Cantu D. A. Wang B. Gongwer M. W. He C. X. Goel A. Suresh A. (2020). EZcalcium: open-source toolbox for analysis of calcium imaging data. *Front. Neural Circuits* 14:25. 10.3389/fncir.2020.00025 32499682 PMC7244005

[B9] Cassidy J. D. Carroll L. J. Peloso P. M. Borg J. Von Holst H. Holm L. (2004). Incidence, risk factors and prevention of mild traumatic brain injury: results of the WHO collaborating centre task force on mild traumatic brain injury. *J. Rehabil. Med.* (43 Suppl) 28–60. 10.1080/16501960410023732 15083870

[B10] Colvin A. C. Mullen J. Lovell M. R. West R. V. Collins M. W. Groh M. (2009). The role of concussion history and gender in recovery from soccer-related concussion. *Am. J. Sports Med.* 37 1699–1704. 10.1177/0363546509332497 19460813

[B11] Corder G. Ahanonu B. Grewe B. F. Wang D. Schnitzer M. J. Scherrer G. (2019). An amygdalar neural ensemble that encodes the unpleasantness of pain. *Science* 363 276–281. 10.1126/science.aap8586 30655440 PMC6450685

[B12] Cossart R. Aronov D. Yuste R. (2003). Attractor dynamics of network UP states in the neocortex. *Nature* 423 283–288. 10.1038/nature01614 12748641

[B13] Creed J. A. Dileonardi A. M. Fox D. P. Tessler A. R. Raghupathi R. (2011). Concussive brain trauma in the mouse results in acute cognitive deficits and sustained impairment of axonal function. *J. Neurotrauma* 28 547–563. 10.1089/neu.2010.1729 21299360 PMC3070143

[B14] Crisco J. J. Fiore R. Beckwith J. G. Chu J. J. Brolinson P. G. Duma S. (2010). Frequency and location of head impact exposures in individual collegiate football players. *J. Athl. Train.* 45 549–559. 10.4085/1062-6050-45.6.549 21062178 PMC2978006

[B15] Frost R. B. Farrer T. J. Primosch M. Hedges D. W. (2013). Prevalence of traumatic brain injury in the general adult population: a meta-analysis. *Neuroepidemiology* 40 154–159. 10.1159/000343275 23257914

[B16] Gabrieli D. Schumm S. N. Vigilante N. F. Meaney D. F. (2021). NMDA receptor alterations after mild traumatic brain injury induce deficits in memory acquisition and recall. *Neural Comput.* 33 67–95. 10.1162/neco_a_01343 33253030 PMC7856344

[B17] Goldstein L. E. Fisher A. M. Tagge C. A. Zhang X. L. Velisek L. Sullivan J. A. (2012). Chronic traumatic encephalopathy in blast-exposed military veterans and a blast neurotrauma mouse model. *Sci. Transl. Med.* 4:134ra160. 10.1126/scitranslmed.3003716 PMC3739428 22593173

[B18] Greco T. Ferguson L. Giza C. Prins M. L. (2019). Mechanisms underlying vulnerabilities after repeat mild traumatic brain injuries. *Exp. Neurol.* 317 206–213. 10.1016/j.expneurol.2019.01.012 30853388

[B19] Guskiewicz K. M. Marshall S. W. Bailes J. Mccrea M. Harding H. P. Jr. Matthews A. (2007). Recurrent concussion and risk of depression in retired professional football players. *Med. Sci. Sports Exerc.* 39 903–909. 10.1249/mss.0b013e3180383da5 17545878

[B20] Hamm J. P. Peterka D. S. Gogos J. A. Yuste R. (2017). Altered cortical ensembles in mouse models of schizophrenia. *Neuron* 94 153–167.e8. 10.1016/j.neuron.2017.03.019 28384469 PMC5394986

[B21] Hansen K. R. Dewalt G. J. Mohammed A. I. Tseng H. A. Abdulkerim M. E. Bensussen S. (2018). Mild blast injury produces acute changes in basal intracellular calcium levels and activity patterns in mouse hippocampal neurons. *J. Neurotrauma* 35 1523–1536. 10.1089/neu.2017.5029 29343209 PMC5998839

[B22] Hsieh T. H. Lee H. H. C. Hameed M. Q. Pascual-Leone A. Hensch T. K. Rotenberg A. (2017). Trajectory of parvalbumin cell impairment and loss of cortical inhibition in traumatic brain injury. *Cereb. Cortex* 27 5509–5524. 10.1093/cercor/bhw318 27909008 PMC6075565

[B23] Kane M. J. Angoa-Perez M. Briggs D. I. Viano D. C. Kreipke C. W. Kuhn D. M. (2012). A mouse model of human repetitive mild traumatic brain injury. *J. Neurosci. Methods* 203 41–49. 10.1016/j.jneumeth.2011.09.003 21930157 PMC3221913

[B24] Koch P. F. Cottone C. Adam C. D. Ulyanova A. V. Russo R. J. Weber M. T. (2020). Traumatic brain injury preserves firing rates but disrupts laminar oscillatory coupling and neuronal entrainment in hippocampal CA1. *eNeuro* 7:ENEURO.0495-19.2020. 10.1523/ENEURO.0495-19.2020 32737188 PMC7477953

[B25] Laker S. R. (2011). Epidemiology of concussion and mild traumatic brain injury. *PM R* 3 S354–S358. 22035677 10.1016/j.pmrj.2011.07.017

[B26] Laurer H. L. Bareyre F. M. Lee V. M. Trojanowski J. Q. Longhi L. Hoover R. (2001). Mild head injury increasing the brain’s vulnerability to a second concussive impact. *J. Neurosurg.* 95 859–870. 10.3171/jns.2001.95.5.0859 11702878

[B27] Lefevre-Dognin C. Cogne M. Perdrieau V. Granger A. Heslot C. Azouvi P. (2021). Definition and epidemiology of mild traumatic brain injury. *Neurochirurgie* 67, 218–221. 10.1016/j.neuchi.2020.02.002 32387427

[B28] Main B. S. Sloley S. S. Villapol S. Zapple D. N. Burns M. P. (2017). A mouse model of single and repetitive mild traumatic brain injury. *J. Vis. Exp.* 124:e55713. 10.3791/55713 28654066 PMC5608469

[B29] McCradden M. D. Cusimano M. D. (2018). Concussions in sledding sports and the unrecognized “sled head”: a systematic review. *Front. Neurol.* 9:772. 10.3389/fneur.2018.00772 30279676 PMC6153360

[B30] McDaid J. Briggs C. A. Barrington N. M. Peterson D. A. Kozlowski D. A. Stutzmann G. E. (2021). Sustained hippocampal synaptic pathophysiology following single and repeated closed-head concussive impacts. *Front. Cell Neurosci.* 15:652721. 10.3389/fncel.2021.652721 33867941 PMC8044326

[B31] Mei Z. Qiu J. Alcon S. Hashim J. Rotenberg A. Sun Y. (2018). Memantine improves outcomes after repetitive traumatic brain injury. *Behav. Brain Res.* 340 195–204. 10.1016/j.bbr.2017.04.017 28412305 PMC5640468

[B32] Miyazaki S. Katayama Y. Lyeth B. G. Jenkins L. W. Dewitt D. S. Goldberg S. J. (1992). Enduring suppression of hippocampal long-term potentiation following traumatic brain injury in rat. *Brain Res.* 585 335–339. 10.1016/0006-8993(92)91232-4 1511317

[B33] Mouzon B. C. Bachmeier C. Ferro A. Ojo J. O. Crynen G. Acker C. M. (2014). Chronic neuropathological and neurobehavioral changes in a repetitive mild traumatic brain injury model. *Ann. Neurol.* 75 241–254. 10.1002/ana.24064 24243523

[B34] Nakajima R. Laskaris N. Rhee J. K. Baker B. J. Kosmidis E. K. (2021). GEVI cell-type specific labelling and a manifold learning approach provide evidence for lateral inhibition at the population level in the mouse hippocampal CA1 area. *Eur. J. Neurosci.* 53 3019–3038. 10.1111/ejn.15177 33675122

[B35] Partridge J. G. Tang K. C. Lovinger D. M. (2000). Regional and postnatal heterogeneity of activity-dependent long-term changes in synaptic efficacy in the dorsal striatum. *J. Neurophysiol.* 84 1422–1429. 10.1152/jn.2000.84.3.1422 10980015

[B36] Patel T. P. Ventre S. C. Geddes-Klein D. Singh P. K. Meaney D. F. (2014). Single-neuron NMDA receptor phenotype influences neuronal rewiring and reintegration following traumatic injury. *J. Neurosci.* 34 4200–4213. 10.1523/JNEUROSCI.4172-13.2014 24647941 PMC3960464

[B37] Prins M. L. Hales A. Reger M. Giza C. C. Hovda D. A. (2010). Repeat traumatic brain injury in the juvenile rat is associated with increased axonal injury and cognitive impairments. *Dev. Neurosci.* 32 510–518. 10.1159/000316800 20829578 PMC3215244

[B38] Ren Z. Iliff J. J. Yang L. Yang J. Chen X. Chen M. J. (2013). ‘Hit & run’ model of closed-skull traumatic brain injury (TBI) reveals complex patterns of post-traumatic AQP4 dysregulation. *J. Cereb. Blood Flow Metab.* 33 834–845. 23443171 10.1038/jcbfm.2013.30 PMC3677112

[B39] Schumm S. N. Gabrieli D. Meaney D. F. (2020). Neuronal degeneration impairs rhythms between connected microcircuits. *Front. Comput. Neurosci.* 14:18. 10.3389/fncom.2020.00018 32194390 PMC7063469

[B40] Sharp D. J. Scott G. Leech R. (2014). Network dysfunction after traumatic brain injury. *Nat. Rev. Neurol.* 10 156–166. 24514870 10.1038/nrneurol.2014.15

[B41] Sloley S. S. Main B. S. Winston C. N. Harvey A. C. Kaganovich A. Korthas H. T. (2021). High-frequency head impact causes chronic synaptic adaptation and long-term cognitive impairment in mice. *Nat. Commun.* 12:2613. 10.1038/s41467-021-22744-6 33972519 PMC8110563

[B42] Tagge C. A. Fisher A. M. Minaeva O. V. Gaudreau-Balderrama A. Moncaster J. A. Zhang X. L. (2018). Concussion, microvascular injury, and early tauopathy in young athletes after impact head injury and an impact concussion mouse model. *Brain* 141 422–458. 10.1093/brain/awx350 29360998 PMC5837414

[B43] Talavage T. M. Nauman E. A. Breedlove E. L. Yoruk U. Dye A. E. Morigaki K. E. (2014). Functionally-detected cognitive impairment in high school football players without clinically-diagnosed concussion. *J. Neurotrauma* 31 327–338. 10.1089/neu.2010.1512 20883154 PMC3922228

[B44] Ting J. T. Daigle T. L. Chen Q. Feng G. (2014). Acute brain slice methods for adult and aging animals: application of targeted patch clamp analysis and optogenetics. *Methods Mol. Biol.* 1183 221–242. 10.1007/978-1-4939-1096-0_14 25023312 PMC4219416

[B45] Villette V. Malvache A. Tressard T. Dupuy N. Cossart R. (2015). Internally recurring hippocampal sequences as a population template of spatiotemporal information. *Neuron* 88 357–366. 10.1016/j.neuron.2015.09.052 26494280 PMC4622933

[B46] Voss J. D. Connolly J. Schwab K. A. Scher A. I. (2015). Update on the epidemiology of concussion/mild traumatic brain injury. *Curr. Pain Headache Rep.* 19:32. 10.1007/s11916-015-0506-z 26049775

[B47] Witkowski E. D. Gao Y. Gavsyuk A. F. Maor I. Dewalt G. J. Eldred W. D. (2019). Rapid changes in synaptic strength after mild traumatic brain injury. *Front. Cell Neurosci.* 13:166. 10.3389/fncel.2019.00166 31105533 PMC6498971

[B48] Wolf J. A. Koch P. F. (2016). Disruption of network synchrony and cognitive dysfunction after traumatic brain injury. *Front. Syst. Neurosci.* 10:43. 10.3389/fnsys.2016.00043 27242454 PMC4868948

[B49] Xia L. Nygard S. K. Sobczak G. G. Hourguettes N. J. Bruchas M. R. (2017). Dorsal-CA1 hippocampal neuronal ensembles encode nicotine-reward contextual associations. *Cell Rep.* 19 2143–2156. 10.1016/j.celrep.2017.05.047 28591584 PMC5524455

[B50] Yuan Q. Isaacson J. S. Scanziani M. (2011). Linking neuronal ensembles by associative synaptic plasticity. *PLoS One* 6:e20486. 10.1371/journal.pone.0020486 21738576 PMC3126801

[B51] Zott B. Simon M. M. Hong W. Unger F. Chen-Engerer H. J. Frosch M. P. (2019). A vicious cycle of beta amyloid-dependent neuronal hyperactivation. *Science* 365 559–565. 10.1126/science.aay0198 31395777 PMC6690382

